# Discovery, design, and engineering of enzymes based on molecular retrobiosynthesis

**DOI:** 10.1002/mlf2.70009

**Published:** 2025-03-28

**Authors:** Ancheng Chen, Xiangda Peng, Tao Shen, Liangzhen Zheng, Dong Wu, Sheng Wang

**Affiliations:** ^1^ Shanghai Zelixir Biotech Company Ltd. Shanghai China

**Keywords:** artificial intelligence, enzyme design, enzyme discovery, enzyme engineering, molecular retrosynthesis planning

## Abstract

Biosynthesis—a process utilizing biological systems to synthesize chemical compounds—has emerged as a revolutionary solution to 21st‐century challenges due to its environmental sustainability, scalability, and high stereoselectivity and regioselectivity. Recent advancements in artificial intelligence (AI) are accelerating biosynthesis by enabling intelligent design, construction, and optimization of enzymatic reactions and biological systems. We first introduce the molecular retrosynthesis route planning in biochemical pathway design, including single‐step retrosynthesis algorithms and AI‐based chemical retrosynthesis route design tools. We highlight the advantages and challenges of large language models in addressing the sparsity of chemical data. Furthermore, we review enzyme discovery methods based on sequence and structure alignment techniques. Breakthroughs in AI‐based structural prediction methods are expected to significantly improve the accuracy of enzyme discovery. We also summarize methods for de novo enzyme generation for nonnatural or orphan reactions, focusing on AI‐based enzyme functional annotation and enzyme discovery techniques based on reaction or small molecule similarity. Turning to enzyme engineering, we discuss strategies to improve enzyme thermostability, solubility, and activity, as well as the applications of AI in these fields. The shift from traditional experiment‐driven models to data‐driven and computationally driven intelligent models is already underway. Finally, we present potential challenges and provide a perspective on future research directions. We envision expanded applications of biocatalysis in drug development, green chemistry, and complex molecule synthesis.

## INTRODUCTION

In today's world, functional molecules play an indispensable role in various aspects of human life, including energy supply, material manufacturing, health maintenance, and medical treatment^1^. However, traditional chemical synthesis methods often rely on fossil fuels, leading to unsustainable resource utilization, environmental pollution, and greenhouse gas emissions^2^. With increasing global emphasis on the philosophy of “green, low‐carbon, and sustainable” production, biosynthesis has emerged as a fundamentally transformative alternative to the “high‐pollution, high‐emission” processing model of chemical synthesis^3^. Particularly in the synthesis of functional molecules with complex chiral centers, biosynthesis offers unparalleled advantages^4^. Enzyme‐based biosynthesis processes provide higher stereoselectivity and regioselectivity, lower costs, and greater efficiency than traditional methods^5^. This selectivity ensures the purity and quality of the product, reduces the complexity and cost of subsequent purification steps, and promises significant economic and environmental benefits.

The ultimate vision of biosynthesis is reflected in the statement “any molecule can be retrobiosynthesized”^6^, ^7^. However, significant challenges remain in achieving this goal, particularly in biochemical pathway design, enzyme discovery and design, and enzyme engineering. The key issues are as follows: (1) Limited accuracy and data availability. Current single‐step retrosynthesis predictions are not sufficiently accurate^8^, ^9^, and databases like UniProt contain incomplete annotations, with only 0.3% of sequences expertly annotated and 19.4% supported by experimental data^10^, ^11^. This restricts the development of many small molecule retrosynthesis pathways. (2) Enzyme discovery challenges. Designing enzymes based on theoretical reaction pathways is difficult due to the insufficient number of characterized enzyme sequences^12^. The orphan reaction problem, where 40%–50% of known enzymatic reactions in databases like KEGG and MetaCyc lack corresponding enzyme sequences^13^, ^14^, ^15^, further complicates enzyme discovery. (3) Gaps in de novo enzyme design. Although de novo enzyme design has shown progress^16^, ^17^, ^18^, ^19^, it still falls short of stable application readiness. (4) Enzyme property optimization. Even with suitable enzymes, their properties such as activity, stability, and substrate specificity often need improvement to meet industrial demands^7^, ^20^, ^21^.

**Figure 1:**
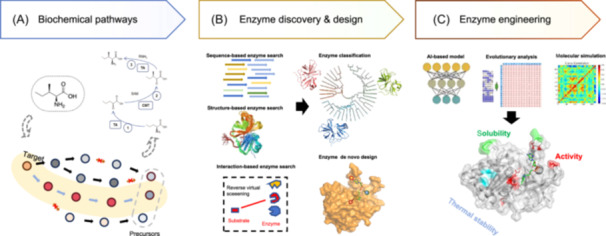
Enzyme design and engineering based on retrosynthetic route planning. (A) Biochemical pathway discovery. The AI‐based retrobiosynthetic route prediction algorithm significantly enhances the discovery efficiency of new reaction pathways. (B) AI‐ and computation‐based multi‐route strategies can increase enzyme discovery efficiency. (C) Fully rational bioelement design methods based on computation aid in the efficient enhancement of element performance.

The development of artificial intelligence (AI) technology in recent years has been remarkable, particularly demonstrating significant potential in the field of biology^22^. A multitude of AI‐based studies have emerged, not only for retrosynthesis but also for enzyme discovery, design, and engineering, which might facilitate the resolution of the aforementioned issues^23^, ^24^, ^25^. Currently, large language models (LLMs) based on the Transformer architecture have played a crucial role in protein sequence modeling^26^, ^27^, ^28^, protein structure prediction^29^, ^30^, ^31^, protein–ligand interactions^32^, ^33^, and protein structure/sequence design^16^, ^34^. The emergence of the Transformer architecture has enabled “pre‐training,” driving the development of various subsequent generative models^35^, ^36^. For example, AlphaFold2 (AF2)^37^ is a groundbreaking work that utilizes the Transformer model to predict biomolecular structures, which are crucial for various biological synthesis designs. In the area of biochemical pathway design, Han et al. utilized a Transformer‐based architecture to effectively improve the accuracy of single‐step retrosynthesis^8^. AlphaFold3^32^ has achieved high‐precision prediction of protein–substrate interactions, which will advance interaction‐based reverse enzyme identification^38^ and enzyme discovery through structure alignment^39^. In de novo enzyme design, numerous generative models have been proposed^12^, ^39^ to generate protein pocket sequences and structures based on protein frameworks and bound small molecules. In the field of enzyme engineering, Transformer‐based predictions and machine learning models can effectively increase the success rate of a variety of enzyme properties^40^, ^41^. These generative models can serve as powerful tools for achieving more precise and efficient biosynthesis (Figure 1).

Given the importance of the aforementioned topics and the rapid advancements in AI disciplines, we believe that it is imperative to discuss the contributions of the latest AI models to biosynthesis. This review proposes new ideas for designing biochemical pathways from scratch. Our objective is to reveal the tremendous potential and future applications of AI technology in enzyme discovery, design, and engineering, providing readers a comprehensive overview and in‐depth analysis.

## DESIGN OF BIOCHEMICAL PATHWAYS

### Molecular retrosynthesis route planning

Molecular retrosynthesis route planning is a key step in biochemical pathway design, through which the synthesis pathway of the target molecule can be identified, providing the necessary prerequisite conditions groundwork for enzyme‐catalyzed reactions^42^. The purpose of this process is to predict potential precursors of a given product through single‐step retrosynthesis algorithms, helping researchers determine the most effective synthesis pathway. In addition to being crucial for the development of industrial strains, this process also forms the basis for enzyme‐directed evolution and the biosynthesis of target substances.

#### Single‐step retrosynthesis algorithms

Single‐step retrosynthesis algorithms are designed to predict potential precursors of a given product through computational methods^43^. These algorithms can be categorized into template‐based methods, template‐free methods, and semi‐template methods^43^ (Table 1).

**Table 1:** Top‐k exact match accuracy on USPTO‐50K.

	Top‐k accuracy (%)							
	Reaction class (unknown)				Reaction class (known)			
Algorithm	1	3	5	10	1	3	5	10
**Template‐based**								
RetroSim	37.2	54.8	63.2	74.2	52.8	73.7	81.2	88.1
NerualSym	44.3	65.3	72.4	78.8	55.3	76.0	81.2	85.1
GLN	52.4	74.6	80.5	86.8	64.1	79.2	85.2	90.1
MHN	50.4	73.8	91.0	87.9	‐	‐	‐	‐
LocalRetro	**53.4**	**77.3**	**85.9**	**92.1**	63.9	86.8	92.4	96.3
DualTB	55.3	74.6	80.4	86.9	67.6	84.8	88.8	92.0
**Semi‐template‐based**								
G2Gs	48.8	67.6	72.4	75.5	61.0	81.3	86.0	88.6
RetroXpert	50.3	61.2	62.3	63.4	62.1	75.6	78.5	80.9
GTA	51.1	67.6	74.8	81.6	‐	‐	‐	‐
GraphRetro	53.6	68.3	72.1	75.5	63.9	81.4	85.2	88.1
MEGAN	48.2	70.7	78.3	86.1	60.7	82.1	87.5	91.6
RPBP	**54.7**	**74.5**	**81.2**	**88.4**	66.6	84.8	90.0	94.5
**Template‐free**								
MT	42.2	61.9	67.4	72.9	54.2	73.6	78.2	81.3
SCROP	43.7	60.0	65.3	68.7	59.0	74.8	78.2	81.1
DMP	46.1	65.3	70.4	74.2	57.5	75.5	80.1	83.1
EditRetro	**60.8**	**80.6**	**86.0**	**90.3**	‐	‐	‐	‐
DualTF	53.6	70.6	74.6	77.0	65.6	81.8	84.7	85.0

Values in bold indicate the top performance for each individual metric in comparative analyses.

Template‐based methods, such as LocalRetro^44^, use local reaction templates to capture the locality of chemical reactions. In most cases, chemical reactions occur within a small portion of the atoms and chemical bonds in a molecule, known as the reaction center. Specifically, LocalTemplate is referred to as an atom template or a bond template depending on whether the reaction center involves atoms or chemical bonds. LocalRetro first generates hidden representations for each node in the input molecular graph using a message‐passing neural network (MPNN) architecture. It then obtains the hidden representation of the edge connecting a pair of connected nodes through a single‐layer fully connected layer. These hidden representations are processed by a global reactivity attention layer and subsequently passed through a fully connected layer to determine the probability of applying a LocalTemplate to each node and edge.

Template‐free methods, such as the EditRetro^8^, include three editing operations, sequence repositioning, placeholder insertion, and label insertion, which are used to generate the reactant strings. EditRetro uses a Transformer architecture, consisting of an encoder and three decoders, all based on stacked Transformer blocks. Extensive tests on the benchmark retrosynthesis dataset USPTO‐50K have shown that EditRetro attains a top‐1 exact match accuracy rate of 60.8%, demonstrating its superior performance.

Semi‐template methods, such as RPBP^45^, first predict potential byproducts of the product molecule and then perform retrosynthesis prediction based on the product and byproducts. RPBP not only considers the potential reaction sites, types, and conditions of byproducts but also enhances the chemical interpretability of the model. However, the main limitation of RPBP is the need for byproduct information during training, which poses a challenge to existing reaction databases. Additionally, generating a large number of potential reactants to pursue higher accuracy and diversity results in a higher proportion of chemically invalid outcomes and significant computational costs.

#### Retrosynthetic route design in chemistry

**Figure 2:**
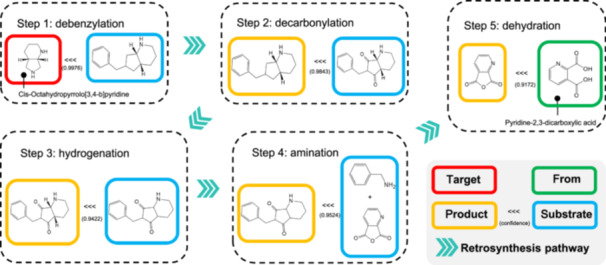
Synthetic pathway of cis‐Octahydropyrrolo[3,4‐b]pyridine via sequential single‐step retrosynthesis synthetic routes of the target molecule. In each retrosynthetic step, the product is listed first, followed by the substrate. These steps are connected sequentially until simpler, known, or commercially available precursor molecules are identified.

In recent years, AI‐based methods have made significant progress in the design of chemical retrosynthesis routes. Figure 2 shows a typical retrosynthesis case using the synthesis of cis‐Octahydropyrrolo[3,4‐b]pyridine as an example. The Retro*^46^ algorithm effectively guides the search for unknown molecules toward more promising directions by leveraging previous design experience, finding high‐quality retrosynthesis routes. Chematica^47^, a software developed by the team of Bartosz A. Grzybowski over more than a decade, systematically links extensive knowledge in organic chemistry. It not only designs multiple synthesis pathways to address raw material shortages but also significantly shortens synthesis routes and reduces costs by optimizing the synthesis of drug molecules and other important chemicals. GNN‐Retro^48^ proposes a new method for estimating the synthesis cost of molecules by applying graph neural networks (GNNs) to obtain synthesis cost information for similar molecules, overcoming data sparsity issues and introducing a semi‐dynamic graph approach to reduce noise during testing, thereby more accurately predicting the synthesis costs of intermediate molecules. AutoSynRoute^49^ uses a heuristic scoring mechanism to evaluate multiple candidate reactions and combines it with the Monte Carlo Tree Search (MCTS) algorithm, effectively searching and combining the optimal reaction routes. Additionally, the AiZynthFinder^50^ algorithm, based on MCTS, selects reaction templates through neural networks and performs iterative searches until it finds purchasable precursors or reaches a predefined maximum depth. It then backpropagates the node scores to initiate the next round of iterations. Unlike Segler's earlier MCTS‐based retrosynthesis planning method, AiZynthFinder does not use filters to exclude unreasonable reactions and adopts a unified strategy during the expansion and rollout phases. Although Synthia^51^ can provide multiple reasonable synthesis routes and literature references for complex compounds, it does not yet offer specific reaction conditions. These research studies and software tools not only enhance the efficiency and accuracy of retrosynthesis planning for compounds but also provide powerful tools for organic chemists.

### Using LLM for retrosynthetic analysis

Recently, the use of LLMs in chemical retrosynthesis tasks has become increasingly widespread. They primarily address the issue of sparse chemical data by learning from available data, thereby enabling more accurate predictions of the synthetic costs and reaction pathways of intermediate molecules. Additionally, LLMs incorporate atomic alignment fusion and distance‐weighted attention modules, enhancing their ability to represent and process complex 3D molecular structures. These models combine multimodal information from sequences, structures, and textual descriptions, making retrosynthesis predictions more comprehensive and accurate. However, due to the structured storage of chemical data, direct use for training may affect the model's performance in natural language processing tasks. Molecular representations, such as SMILES sequences, do not conform to natural language norms, making it difficult for conventional LLMs to process and generate them. Moreover, the variety of chemical tasks and data types poses significant challenges in building a training framework that can be broadly applied to different chemical tasks.

In response to the challenges mentioned above, the Shanghai Artificial Intelligence Laboratory developed ChemLLM^52^, which performs comparably to GPT‐4^53^ in core chemical tasks while also demonstrating excellent capabilities in general conversation and natural language processing tasks. ChemLLM is based on the InternLM2‐Base‐7B model and is trained using a two‐stage instruction fine‐tuning method, achieving enhanced chemical capabilities. In terms of retrosynthesis prediction, ChemGPT 2.0^54^ adopts a template‐free retrosynthesis prediction model, integrating 3.78 million high‐quality retrosynthesis data points and 3D molecular conformation information. By introducing atomic alignment fusion and distance‐weighted attention modules, it provides significant improvements in the accuracy of molecular representation. ChemGPT 2.0 not only performs well on benchmark datasets but can also more accurately predict the reactants of complex 3D structured molecules. Furthermore, multimodal language models such as ChemVLM^55^ have further advanced the development of retrosynthesis tasks. ChemVLM combines the advantages of visual Transformers (ViTs), multilayer perceptrons (MLPs), and LLMs, utilizing pre‐trained ChemLLM‐20B for chemical text understanding and InternVIT‐6B for image feature extraction, enabling comprehensive reasoning on both chemical images and text. By integrating these technologies, ChemVLM provides more comprehensive and accurate predictions when handling retrosynthesis tasks.

Although LLMs have made significant progress in chemical retrosynthesis tasks, there is still a need for more comprehensive benchmark tests to evaluate their performance in advanced chemical concepts and tasks. ChemEval^56^ has been developed for this purpose, providing a series of multi‐level tasks to comprehensively assess the processing and application capabilities of large models in the chemical domain, ranging from basic chemical questions to complex molecular structure analysis, chemical reaction prediction, and scientific knowledge inference.

## ENZYME DESIGN BASED ON BIOCHEMICAL PATHWAYS

Enzymes are a class of biomacromolecules with high specificity and catalytic efficiency. Discovery of new enzymes can lead to improved production efficiency, reduced costs, and minimized environmental pollution^57^. For example, certain proteases and amylases have been widely used in the food industry for the breakdown of proteins and starch. With advancements in genomics and proteomics, researchers can predict and identify potential new enzymes through comparative analysis^58^. The development of computational biology and bioinformatics has provided powerful tools for enzyme discovery^59^. Through computer simulations and data analysis, the structure and function of enzymes can be predicted, guiding experimental design^60^.

### Enzyme discovery using sequence alignment

In enzyme discovery, sequence search is a critical step for uncovering the evolutionary relationships, conserved regions, and functional characteristics of the target enzyme^61^. Sequence alignment techniques are primarily used to determine the arrangement that produces the highest similarity score between two or more sequences, typically based on dynamic programming algorithms, such as the Needleman–Wunsch algorithm^62^. Through sequence alignment, insertions and deletions in DNA sequences can be identified, revealing conserved and non‐conserved regions and providing insights into evolutionary trends. Sequence alignment can be divided into pairwise alignment and multiple alignment. Concepts that describe the relationships between sequences include homology, similarity, and distance. Homology is a qualitative concept, indicating that different sequences share a common evolutionary ancestor; similarity and distance are quantitative concepts for measuring the similarities and differences between sequences. Homologous sequences usually have high similarity, but high similarity does not necessarily imply a homologous relationship, as convergent evolution can also lead to different sequences from different sources showing similarity.

In enzyme discovery using sequence alignment methods, Skolnick et al. introduced the EFICAz method^63^, a comprehensive approach for large‐scale enzyme function inference, which integrates protein, genomic, and metagenomic databases to discover new enzymes and metabolic pathways. Copp et al.^64^ revealed an unexplored sequence–function space using SSNs, which leverages SSNs to infer protein functions and provides new insights into enzyme discovery and functional annotation. Atkinson et al.^65^ used SSNs to visualize the relationships between different protein superfamilies, emphasizing the value of SSNs in revealing the diversity and functional distribution within protein families. Barber et al. developed the Pythoscape framework^66^ for generating large protein similarity networks. This tool aids researchers in exploring the functional diversity and evolutionary relationships within protein families. Gerlt et al. have introduced the EFI network resource^67^, a tool for genomic enzymology that utilizes protein, genomic, and metagenomic databases to discover new enzymes and metabolic pathways.

Multiple sequence alignment (MSA) tools, such as MAFFT^68^, overcome the limitations of progressive MSA algorithms by iteratively aligning and optimizing sequences, thereby improving the accuracy and total score of multiple sequence alignments. Additionally, the FastMSA framework^69^ significantly enhances the scalability and speed of multiple sequence alignments through the combination of a query sequence encoder and a context sequence encoder. This framework can achieve a 93‐fold acceleration in processing large‐scale sequence databases, substantially reducing the search time of JackHMMER.

At the same time, tools based on protein language models (PLMs), such as THPLM^70^, are also gaining prominence. These tools require only sequences as input and can uncover remote homology information hidden within sequences through deep learning models and Pfam sequence analysis, thereby improving the sensitivity and convenience of homologous protein searches. Given that sequence data are easier to obtain and apply than structural data, these tools have broad application prospects in enzyme discovery.

Many excellent review articles have emerged in the field of sequence alignment for enzyme design. Zaparucha et al. provided a detailed overview of how genome mining techniques can aid in the discovery of new enzymes by leveraging genomic data to identify potentially active enzymes^71^. Wang et al. reviewed a series of computational tools specifically implemented for the design and reconstruction of metabolic pathways, which predict and annotate enzyme functions by analyzing sequence similarity^72^. Saa further explores the application of these tools in yeast systems and synthetic biology, emphasizing their importance in enzyme discovery and functional annotation using bioinformatics methods^73^. Scherlach et al. underlined the biosynthetic potential through genome mining, including the identification of key enzymes in plants and the discovery of new biosynthetic pathways, demonstrating the utility of bioinformatics tools and SSNs in the discovery of new enzymes and biosynthetic pathways^74^. Collectively, these review articles highlight the critical role of sequence alignment techniques in enzyme design and the exploration of biosynthetic pathways.

### Enzyme discovery using structure alignment

**Figure 3:**
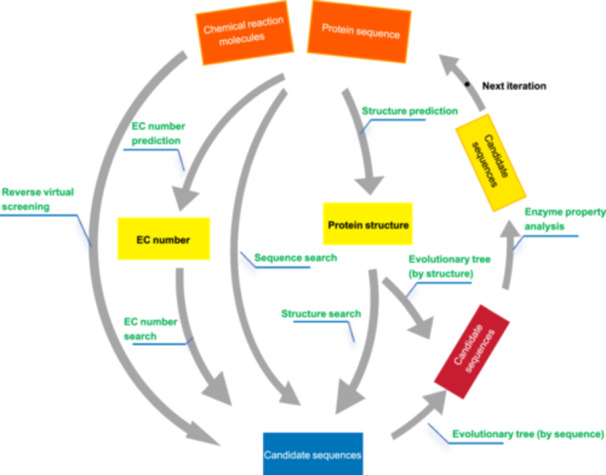
Enzyme discovery via multiple strategies for enhanced success rates. Enzyme discovery is performed using multiple approaches, including sequence search, structure search, EC (Enzyme Commission) number prediction and retrieval, and reverse virtual screening. The first two methods use the initial enzyme as a starting point, while the latter two begin with the reaction or molecule. These strategies can be used individually, in combination, or iteratively to increase the likelihood of successful enzyme discovery.

The three‐dimensional structure of enzymes is a critical determinant of their catalytic function and activity^75^. In the genomic era, significant achievements have been made in uncovering protein functions and evolutionary insights through sequence analysis. However, protein sequences typically mutate faster during evolution than their structures, making sequence‐based analyses less effective in highly divergent proteomes. Therefore, extracting implicit information directly from structures has become a more effective strategy^76^. Recent advancements in 3D structures achieved through experiments and breakthroughs in protein structure prediction using AI‐based technologies, such as AlphaFold2, have significantly improved the efficiency and scale of novel protein discovery^37^. The application of these technologies has made structure‐based functional protein discovery more efficient and accurate (Figure 3).

In terms of structure search tools, Muscle‐3D^77^ leverages a “mega‐alphabet” to represent structural information, introduces posterior decoding for aligning hidden Markov models (HMMs), and uses an iterative optimization strategy. This approach helps capture structural information more accurately and improve the stability and precision of handling large datasets. Foldseek‐Multimer^78^ has achieved a significant speedup (three to four orders of magnitude) in protein complex structure analysis while maintaining high accuracy, allowing the discovery of structural homologies with very low sequence similarity. Additionally, Tyzack curated the FunTree database^79^, which compiles sequence, structure, phylogenetic, chemical, and mechanical information for 2340 CATH superfamilies (each containing at least one enzyme). FunTree uses sequence and structure alignments to cluster proteins within superfamilies into structurally similar groups (SSGs) and generates phylogenetic trees enhanced through ancestral feature estimation (ACE).

The development of these tools and databases has enabled structure search to be more widely applied in enzyme discovery, providing richer functional and evolutionary information. Xu et al.^80^ predicted the 3D structures of 1483 cytidine deaminases using AlphaFold2 and performed structural similarity clustering. They ultimately selected representative enzymes for experimental characterization, identifying some deaminases with high editing efficiency and diversity. Huang et al.^81^ used structural modeling and similarity alignment to investigate unknown characteristics of the deaminase protein family. They found that most proteins in the DddA‐like branch are not double‐stranded DNA deaminases and discovered a deaminase that can efficiently edit soybean plants. Chen et al.^82^ combined structural similarity search and sequence‐based protein clustering to discover a novel tagatose 4‐epimerase (Thar‐T4Ease) from the archaeon *Thermoprotei*, which can convert d‐fructose into d‐tagatose. Deng et al.^83^ identified several small single‐stranded DNA deaminases (Sdds) using a sequence alignment‐based method (WFG strategy). Through structural modeling, they identified conserved regions in the structure, effectively reducing the size of Sdds while maintaining their efficiency. Feng et al.^84^ successfully identified the flavonoid glycosyltransferase NjUGT73B1 by clustering predicted structural similarities. This enzyme can effectively catalyze the glycosylation at the 7‐OH position of acacetin, which is a key precursor in linarin biosynthesis.

### De novo generation of enzymes based on reaction pathways

For nonnatural reactions or orphan reactions, there may not be an initial enzyme available. In such cases, it is not possible to search for enzymes using sequence or structure similarity. Instead, proteins that potentially match the target reaction can be found by annotating the functions of protein libraries through reaction similarity, small molecule similarity, enzyme–substrate interaction predictions, or de novo enzyme generation^85^.

#### AI‐based enzyme function annotation

Significant advances have been made in AI‐based enzyme function annotation techniques in recent years. Yu et al. introduced the CLEAN (Contrastive Learning–Enabled Enzyme Annotation) algorithm^11^, which accurately predicts Enzyme Commission (EC) numbers through contrastive learning. This algorithm has shown exceptional performance in annotating enzyme functions, correcting erroneous EC numbers, and identifying multifunctional enzymes. Additionally, Kandlinger et al. developed the AGeNNT tool^86^, which helps distinguish the functional coupling of enzymes inferred from a large number of phylogenetically distant species by analyzing the complexity of genomic neighborhoods (GNs) and refined genomic neighborhoods (rGNs), demonstrating the application of GN networks in enzyme function inference.

Shi et al. developed a deep learning‐based algorithm called HDMLF (Hierarchical Dual‐core Multitask Learning Framework)^87^, achieving high accuracy and reliability in protein function prediction. The team also launched a free public protein function annotation platform called ECRECer, which can annotate newly discovered proteins as enzymes or non‐enzymes, annotate the functions of promiscuous enzymes, and correct or complete the annotations of enzymes with incomplete or incorrect annotations. Yang et al. proposed the CLEAN‐Contact framework^88^, which combines contrastive learning of protein amino acid sequences and contact maps, significantly enhancing the accuracy of enzyme function prediction and enabling the identification of novel enzyme functions within the *Prochlorococcus marinus* MED4 proteome.

Liang et al. introduced a method that directly utilizes the relationships between amino acids to construct a structural relationship network (SRN), achieving a classification accuracy of 92.08% on large datasets^89^. Song et al. proposed GraphEC^90^, which is a geometric graph learning‐based EC number predictor. GraphEC leverages ESMFold for structure prediction and pre‐trained PLMs to predict enzyme active sites and EC numbers, further improving the results through homology information and label propagation algorithms, and simultaneously predicting the optimal pH of enzymes^90^. Zheng et al. developed the AnnoPRO strategy^91^, which implements multi‐scale protein representation based on sequences, uses pre‐trained dual‐path protein encoding, and performs function annotation using long short‐term memory (LSTM) decoding. Case studies based on different benchmarks have confirmed the superior performance of AnnoPRO compared to existing methods^91^. These methods collectively advance the precision and efficiency of enzyme function annotation.

#### Enzyme discovery based on reaction similarity or small molecule similarity

In the absence of initial enzyme clues, enzyme discovery methods based on reaction similarity or small molecule similarity become effective alternatives. The EC‐BLAST algorithm^92^ and web tool perform quantitative similarity search of enzyme reactions at three levels, i.e., bond changes, reaction centers, and reaction structural similarity, demonstrating its potential in enzyme classification, and identifying new reactions, enzyme function assignment, and enzyme engineering. E‐zyme2^93^ searches for similar substrate–product pairs in reference databases using the chemical structures of substrate–product pairs, enabling the identification of orthologous gene clusters that may mediate a given reaction. The Selenzyme tool^94^ not only calculates reaction similarity but also provides information on phylogenetic distance, conserved regions, predicted catalytic sites, and active regions, as well as solubility or transmembrane regions, supporting the design of metabolic pathways. Plehiers et al. developed a method for extracting reaction templates from chemical databases, which is based on correct atom mapping and is applicable to various reaction types^95^. Probst proposed a data‐driven human–machine interaction machine learning approach that automates the association of catalytic enzymes with given biochemical reactions, a key step in linking reaction similarity to enzyme localization^96^. The REME platform^97^, combines atom‐atom mapping, atom‐type change recognition, and reaction similarity calculations to quickly rank and visualize enzyme reactions similar to the target nonnatural reaction, and allows users to filter or expand results by functional groups, species, and EC numbers. The EnzFIND method^98^ identifies all enzymes that can catalyze a reaction based on the molecular feature similarity between the enzyme's natural reaction and probe reaction, achieving a maximum accuracy of 0.95 and successfully predicting 112 reactions not included in the *E. coli* metabolic model. Martínez Cuesta proposed a method for describing isomerases, enhancing the efficiency of reaction data search^99^. The Transform‐MinER tool^100^ facilitates the conversion of substrate molecules into products via known enzyme reactions at potential reaction centers, identifying potential enzyme reactions, or attempting to link source and target molecules with enzyme. Additionally, specialized tools like RxnSim^101^ and SimCAL^102^ are available. The former considers molecular features when calculating reaction similarity, while the latter incorporates novel physicochemical features such as stereochemistry, mass, and volume, both performing well in reaction similarity calculations. The Schwaller team used a Transformer‐based model to infer reaction categories from simple text‐based chemical reaction representations, achieving a classification accuracy of 98.2%^103^. The differential reaction fingerprint (DRFP) algorithm^104^ excels in reaction yield prediction and reaction classification tasks, achieving state‐of‐the‐art performance. These methods and tools provide significant support for enzyme discovery and metabolic engineering.

#### Enzyme databases

Enzyme databases play a crucial role in biochemical research by expanding and deepening the understanding of enzymes and their catalytic reactions through various strategies (Table 2). EnzyMine^105^ enhances the connection between enzymes and metabolic reactions by integrating reaction chemical feature strategies, focusing on the description of enzyme reaction characteristics and linking them with sequence and structure annotations. This approach has the potential to reveal many new metabolic pathways associated with specific enzymes, thereby expanding the functional annotation of enzymes. BRENDA^106^ is a comprehensive enzyme information system that integrates a large amount of enzyme data from the literature, including enzyme classification, chemical properties, function, gene sequences, expression information, known substrates and products, specific inhibitors and activators, effectors, *K*_m_ (Michaelis constant) values, and the optimal ranges of temperature and pH.

**Table 2:** Overview of enzyme reaction databases.

Database	Features	Website
KEGG	Containing biological pathways, compounds, genes, and proteins	https://www.kegg.jp/kegg
UniProt	UniProt stands as the world‐leading repository, offering an extensive, high‐quality, and openly accessible database of protein sequences, structures, and functional data	https://www.uniprot.org/
PDBe	As a founding member of the Worldwide Protein Data Bank (wwPDB), PDBe plays a pivotal role in the collection, organization, and dissemination of critical biological data	https://www.ebi.ac.uk/pdbe/
IntEnz	Containing data on enzymes organized by enzyme EC number and is the official version of the Enzyme Nomenclature	https://www.ebi.ac.uk/intenz/index.jsp
ChEBI	A comprehensive, open‐access repository dedicated to cataloging molecular entities, with a particular emphasis on ‘small’ chemical compounds	https://www.ebi.ac.uk/chebi/
ChEMBL	A manually curated repository of bioactive molecules with drug‐like characteristics, integrating chemical, bioactivity, and genomic data into a unified resource	https://www.ebi.ac.uk/chembl/
MetaCyc	Containing information on the metabolic pathways of various organisms	https://metacyc.org
Rhea	Containing relationships between reactions, enzymes, and compounds	https://www.rhea‐db.org
BRENDA	A database dedicated to enzyme and metabolic reaction information	https://www.brenda‐enzymes.org
SABIO‐RK	A database focused on the kinetics of biochemical reactions	https://sabiork.h‐its.org
Reactome	An open knowledge base focused on metabolic pathways and signal transduction	https://reactome.org
PathBank	A database dedicated to metabolic pathways	http://www.pathbank.org
HMDB	A database focused on human metabolic products	https://hmdb.ca
MetaNetX	A database focused on metabolic networks	https://www.metanetx.org
ExPASy‐ENZYME	It is fundamentally guided by the guidelines and recommendations established by the Nomenclature Committee of the International Union of Biochemistry and Molecular Biology	https://enzyme.expasy.org/
ExPASy‐PROSITE	Database of protein domains, families, and functional sites	https://prosite.expasy.org/
Protein Data Bank	As a member of the wwPDB, the RCSB PDB curates and annotates PDB data according to an agreed‐upon standard	https://www.rcsb.org/
FireProtDB	A meticulously curated and comprehensive repository of protein stability data, specifically focusing on single‐point mutations	https://loschmidt.chemi.muni.cz/fireprotdb/
BioCatNet	Database system aims to collect and present comprehensive information about biocatalysts: sequence, structure, educts, and products	https://www.biocatnet.de/
Reaxys	A database covering the fields of chemistry, pharmaceuticals, and materials science	https://www.reaxys.com
EnzyMine	The incorporation of chemical structural data into enzymatic reaction analysis has demonstrated substantial importance in enhancing the precision of enzyme function prediction	http://www.rxnfinder.org/enzymine/

EnzymeMap^107^ integrated a large toolbox for automatic reaction management and correction steps, resulting in high‐quality atom mapping that includes stereochemical information and a balanced dataset of enzyme reactions. This toolbox has been applied to the entries of natural and nonnatural substrate–product pairs in BRENDA. The Enzyme Portal^108^ serves as an interface, providing enzyme data from the European Bioinformatics Institute (EMBL‐EBI), including enzyme function, sequence, structure, family, substrate, reactions, pathways, diseases, and related literature. The data sources include the UniProt^10^ knowledge base (UniProtKB), the European Protein Database (PDBe^109^), Rhea^110^—a database of enzyme‐catalyzed reactions, Reactome^111^—a database of biochemical pathways, IntEnz^112^—a resource for enzyme nomenclature information, and ChEBI^113^ and ChEMBL^114^—resources containing chemical and biological activity information of small molecules.

#### Inverse virtual screening (IVS) based on small molecule–target interactions

**Figure 4:**
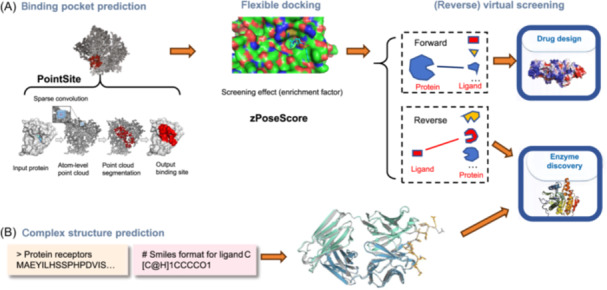
Enzyme discovery based on protein–ligand interactions using traditional and AI‐driven approaches. (A) Traditional bioinformatics methods using PointSite and zPoseScore for applications in drug design and enzyme discovery. (B) Direct structure prediction methods, such as AlphaFold3, leveraging AI to predict protein structures from amino acid sequences.

IVS technology, based on interactions between small molecules and targets, has shown significant application value in enzyme discovery and drug target discovery (Figure 4). By analyzing the interactions between enzymes and small molecule substrates, IVS can not only identify potential targets for known ligands or crystal structures but also screen for new binding proteins from a large number of receptors. For example, Do et al. used docking programs to determine the binding targets of two natural products: ε‐viniferin and meranzin. From a manually collected database containing 400 targets, they identified cyclic nucleotide phosphodiesterase 4 (PDE4) as the target for ε‐viniferin, while COX1, COX2, and PPARγ were found to be the targets for meranzin^115^, ^116^, ^117^. Additionally, Slon‐Usakiewicz et al. combined ultra‐sensitive mass spectrometry with docking‐based IVS to explore the mechanism of action of methotrexate (MTX)^118^. They discovered that besides the three known primary targets, namely, dihydrofolate reductase, thymidylate synthase, and glycylamidinoribonucleotide transformylase, at least eight other proteins were recognized as possible MTX targets. The authors used affinity chromatography coupled with mass spectrometry to further validate one of the predicted targets, hypoxanthine–guanine phosphoribosyltransferase (HGPRT), as a true binding partner of MTX, with a Kd value of 4.2 μmol/l.

Huang et al. primarily discussed inverse screening methods for identifying protein targets of chemical preventive compounds or drugs^119^. These compounds include traditional Chinese medicine components, natural compounds, and FDA‐approved drugs. While these compounds show a certain degree of selectivity for specific targets, they frequently bind to diverse receptors across various signaling pathways within human cells. Unlike traditional virtual screening, inverse screening methods, also known as computational target fishing^120^, have the advantage of identifying a wider range of potential or unexpected targets for a specific compound. This is achieved by analyzing known binding information or predicting receptor–ligand affinity, allowing for a more exhaustive exploration. The applications of this approach are extensive. It can identify the potential receptors of query molecules derived from natural products of terrestrial or marine, find new therapeutic uses for existing drugs through drug repositioning, and explore the chemopreventive compounds mechanisms.

In a review article, Xu et al. provided a detailed introduction to inverse virtual screening (IVS) technology^121^, a computational method used to identify potential protein targets of small molecule ligands, which is crucial for the drug development process. Their article not only explains the basic principles of IVS technology but also demonstrates its applications in drug discovery and optimization through multiple case studies. Another review article by Agu et al. explores the application of molecular docking technology in nutraceutical research and disease management^122^. Molecular docking technology, by simulating the interactions between small molecules and proteins, provides a powerful tool for identifying potential targets of nutraceuticals, contributing to the development of new strategies for disease prevention and treatment. These studies not only advance the identification of enzyme targets but also provide important computational tools and experimental methods through in‐depth exploration of drug mechanisms of action and drug repositioning, thereby facilitating enzyme discovery.

#### De Novo enzyme design protocol

In the field of enzyme design, the use of PLMs is becoming increasingly widespread. These models draw on self‐supervised learning techniques from natural language processing and, by training on large amounts of protein sequence data, they can capture evolutionary, structural, and functional information of proteins, thereby providing strong support for enzyme design. For instance, ESM Cambrian is a parallel series of the ESM3 generative model released by EvolutionaryScale, focusing on creating biological representations of protein sequences^28^. By expanding the data and computational resources for training, its performance has been significantly enhanced compared to ESM2. The FSFP (Few‐Shot Fine‐tuning for Proteins) method^123^ integrates meta‐learning, learning to rank, and parameter‐efficient fine‐tuning, enabling a substantial improvement in the accuracy of protein mutation‐property predictions even with limited wet‐lab experimental data. In the ProteinGym test, which includes 87 high‐throughput mutation datasets^124^, FSFP evaluated the target protein against the two most similar protein datasets in the collection and combined GEMME scoring data. Ultimately, the model trained on just 20 wet‐lab experimental data points improved the predicted Spearman correlation from below 0.1 to over 0.5.

Furthermore, the Progen model^125^ introduces a large number of control labels, enabling it to train on amino acid sequences and to generate protein sequences with specific functions. Researchers selected 100 proteins for testing based on the naturalness of their amino acid sequence semantics and syntax. The results showed that 72% of the proteins were well expressed, and even when the differences from natural proteins increased, the quality of expression of the artificial proteins remained comparable to that of natural proteins. The application of these models not only enhances the accuracy of protein function prediction but also opens up new possibilities for enzyme design.

In recent years, the emergence of various enzyme tools has made de novo enzyme design pathways feasible. These pathways typically start with a given chemical reaction and utilize bioinformatics tools such as EC‐Blast^92^ to precisely identify the EC number of the enzyme required to catalyze the reaction. EC‐Blast identifies known enzyme reactions similar to the query reaction by comparing bond changes, reaction centers, and structural similarities, thereby aiding in the assignment of EC numbers. Once the EC number is determined, conditional PLMs like ZymCTRL^126^ can be used to generate the corresponding enzyme sequences. ZymCTRL has been trained on 37 million enzyme sequences classified by EC number from the publicly available BRENDA database. By learning the specific sequence features of each catalytic reaction, ZymCTRL can efficiently generate the required enzyme sequences.

**Figure 5:**
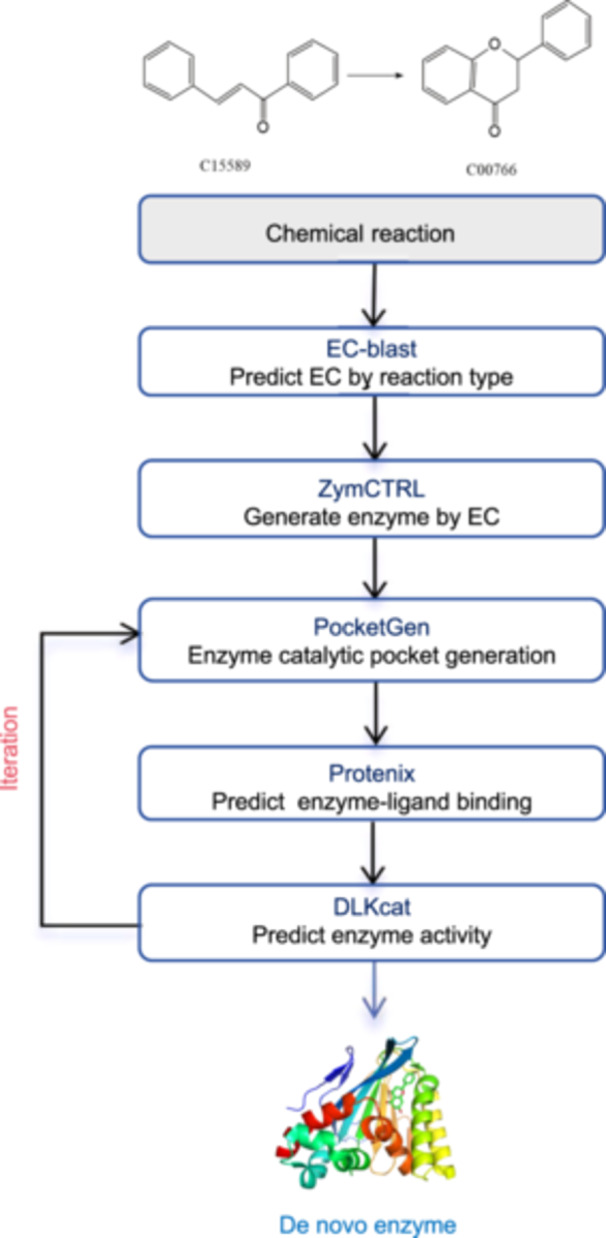
De novo enzyme generation and optimization based on reaction pathways. Starting from the chemical reaction, the sequential steps involved in enzyme generation and optimization: predicting the EC number using EC‐blast; generating the enzyme with tools like ZymCtrl; redesigning the catalytic pocket for enhanced small molecule binding using PocketGen and similar tools; predicting protein conformation with Protenix and analogous methods; and assessing enzyme activity via DLKcat and other predictive approaches. If necessary, the catalytic pocket is regenerated and re‐evaluated iteratively until the desired performance is achieved.

To further optimize enzyme design, tools such as AlphaFold3^32^ and Protenix can be utilized to predict the structure of the substrate–enzyme complex, ensuring proper binding of the substrate to the enzyme through structural prediction. Subsequently, tools like PocketGen^39^ or EnzymeFlow^12^ can be used to generate the catalytic pockets of the enzyme. PocketGen uses a combination of a two‐layer graph Transformer and a protein language model, using a multi‐layer attention mechanism to capture the geometric shapes and interactions at various layers of protein–ligand complexes, thereby generating and optimizing catalytic pockets. EnzymeFlow, on the other hand, is based on a generative model using flow matching, which captures the dynamic interactions between enzymes and substrates during the evolutionary process through a hierarchical pre‐training strategy on protein backbones, protein–ligand complexes, and enzyme‐reaction datasets, generating enzyme catalytic pocket structures capable of catalyzing the target reaction (Figure 5).

Finally, the activity of the generated enzymes can be predicted using tools like DLKcat, which combines GNNs and convolutional neural networks (CNNs) to capture *K*_cat_ changes in mutant enzymes and identify amino acid residues that significantly impact enzyme activity, aiding in the modification and optimization of enzymes. Additionally, common enzyme design tools such as PocketFlow^127^, RFDiffusion AA^17^, and ProteinMPNN^34^ can generate sequences while keeping the protein backbone unchanged, thereby improving the solubility and stability of the proteins. The comprehensive application of these tools and methods has made de novo enzyme design pathways widely used and developed in enzyme engineering.

## ENZYME ENGINEERING

**Figure 6:**
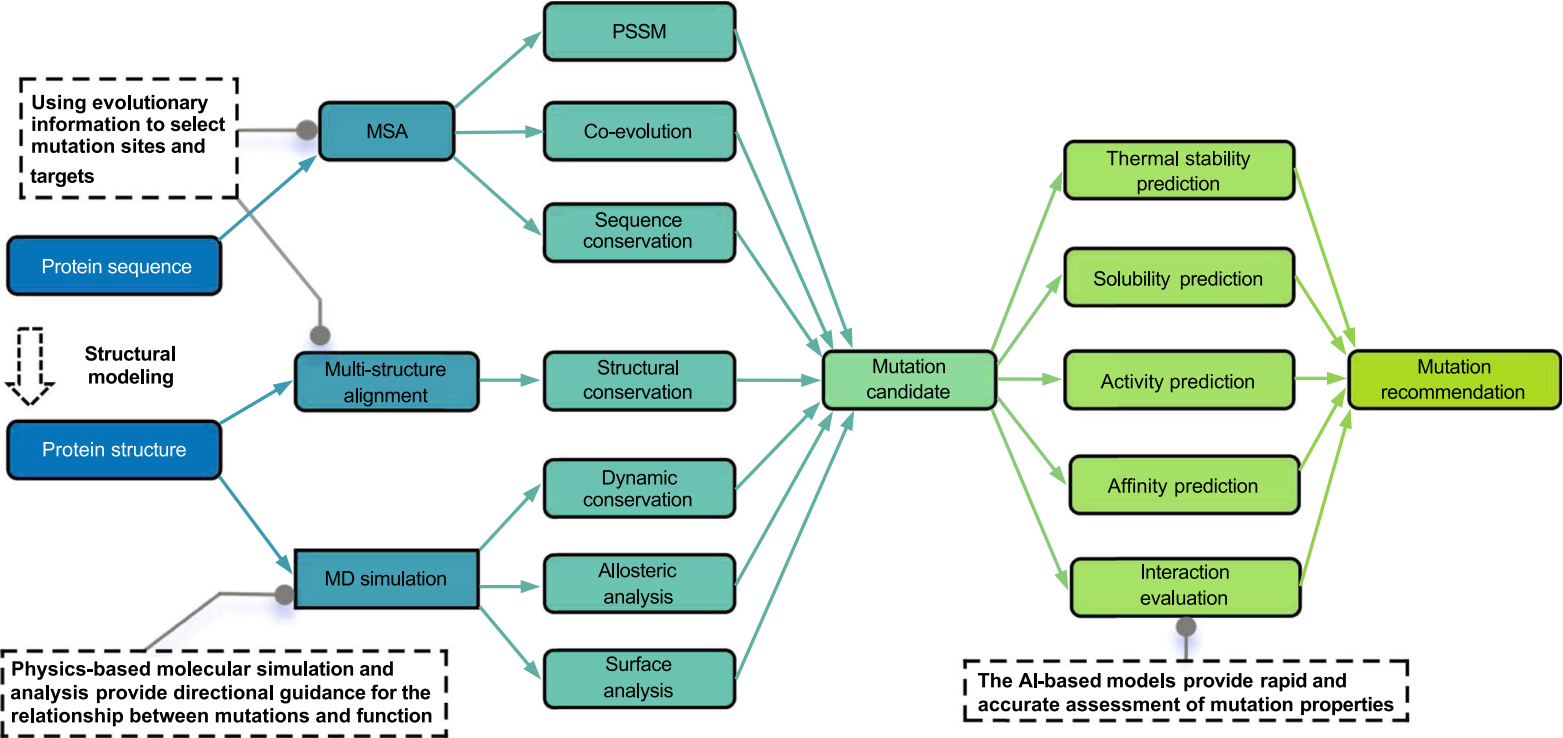
Typical enzyme engineering pathways. Protein sequence analyses, such as multiple sequence alignment (or multiple structure alignment, MSA), coevolution, position‐specific scoring matrix (PSSM), and conservation calculations, can be used to help select mutation sites and targets. Molecular Dynamics (MD) can be used to provide mutation guidance, such as catalytic mechanism, near‐attack state, and motion correlation between residues. AI‐based models can be used to rapidly and accurately assess properties of mutations, including thermal stability, solubility, enzyme activity, binding affinity, and interaction evaluations.

Enzyme engineering typically focuses on enhancing aspects such as thermal stability, solubility, activity, and affinity^128^ (Figure 6). Through these modifications, enzymes can show superior performance in industrial applications, making them more adaptable to complex industrial environments. These improvements not only enhance the efficiency of enzymes but also broaden their potential applications in various manufacturing processes.

In the field of enzyme engineering, the application of AI tools is becoming increasingly widespread^129^. These tools provide strong support for the directed evolution and rational design of enzymes through advanced algorithms and big data analysis. AI technologies can process and analyze vast amounts of biological data, including enzyme structure, function, evolutionary history, and performance under different conditions, thereby helping researchers make more precise predictions and optimizations of enzyme performance^130^. The introduction of AI technology has significantly expanded the boundaries of enzyme engineering, shifting the field from a traditional experiment‐driven approach to a data‐ and computation‐driven intelligent approach.

Traditional biophysical computational methods, such as evolutionary analysis and molecular dynamics simulations, also play a significant role in enzyme engineering and rational design. Therefore, alongside AI‐based approaches, representative applications of these methods will be briefly discussed in subsequent sections. Since this review focuses on AI‐based methods, we will not go into detail on this topic. Some reviews present a good summary^131^, ^132^, ^133^.

### Thermal stability engineering of enzymes

The engineering of enzymes for thermal stability is a critical aspect of their application in industry. However, a common challenge is that improving thermal stability often leads to a decrease in enzyme activity^134^. To find a balance between thermal stability and enzyme activity, researchers have used various protein engineering methods, including directed evolution, semi‐rational design, and rational design. These methods are used to modify enzymes at both the genetic and structural levels. Strategies such as increasing substrate affinity, introducing electrostatic interactions, eliminating steric hindrance, enhancing flexibility at the active site, and N‐terminal and C‐terminal engineering are widely applied to improve both the thermal stability and activity of enzymes. For example, enhancing hydrophobic interactions within the molecule and introducing proline residues have been shown to improve thermal stability without significantly reducing activity.

Recently, the team led by Professor Bin Yao and Dr. Tao Tu from the Chinese Academy of Agricultural Sciences, in collaboration with Dr. Haobo Wang's team from Hangzhou Liewen Research Institute, proposed the “weakest link” theory and validated it using a “zero‐shot Hamiltonian model” (ZSH)^135^. This theory analogizes proteins to a wooden bucket, where each component represents a board, and the overall thermal stability of the enzyme depends on the least stable part of its structure. Using α‐amylase, an important industrial enzyme, as an example, they demonstrated the existence of the “weakest link” and its impact on thermal stability through domain swapping at different levels. The experimental results showed that by swapping the B domain of the thermostable α‐amylase (thermoAMY) into the mesophilic α‐amylase (mesoAMY), the *T*_m_ value of the chimeric mesoAMY‐B was significantly increased by 12°C, indicating that repairing the least stable B domain can significantly enhance the enzyme's thermal stability. Conversely, swapping the B domain of mesoAMY into thermoAMY resulted in a decrease in the *T*_m_ value of thermoAMY‐B, further confirming the “weakest link” characteristic of the B domain in terms of thermal stability.

Additionally, deep learning‐based PLMs such as Pro‐PRIME^136^ and ProtREM^137^ have shown great potential in thermal stability engineering. The Pro‐PRIME model is trained using a “temperature‐aware” language model, enabling it to predict performance improvements of specific protein mutants without relying on experimental data. The ProtREM model, by integrating sequence, structural, and evolutionary information, not only accurately predicts mutation effects but also guides the design of superior mutants. The introduction of these models provides a new approach to thermal stability engineering, significantly reducing the time and cost associated with screening mutant libraries.

### Solubility engineering of enzymes

Solubility engineering of enzymes is a crucial research direction in the field of protein engineering, aimed at meeting specific catalytic requirements and ensuring that enzymes function more stably and effectively in aqueous environments. Many natural enzymes can lose their activity in aqueous environments due to high temperatures, pH changes, exposure to organic solvents, or prolonged storage^138^. Through solubility engineering, the stability and activity of enzymes under various conditions can be significantly enhanced, prolonging their lifespan in practical applications^5^. In certain biochemical reactions, the solubility of enzymes is a key limiting factor.

By adjusting the surface charge distribution, hydrophilicity, and hydrophobicity of enzymes, their solubility in water can be significantly improved, leading to increased reaction rates and yields. For example, a collaborative team from Shanghai Jiao Tong University and the Massachusetts Institute of Technology (MIT) spent 7 years developing the QTY code^139^, a method specifically designed for protein engineering, particularly for improving the solubility of membrane proteins. In the QTY code, Q represents glutamine (Gln), T represents threonine (Thr), and Y represents tyrosine (Tyr). The goal of this method is to transform proteins that are originally insoluble in water into water‐soluble forms while maintaining their native conformation and biological function. Compared to traditional methods, the QTY code has two significant advantages: it is simple and easy to operate without relying on complex computer programs and it enables direct design of protein sequences without requiring pre‐existing structural data.

Proteins designed using the QTY code, such as the membrane protein CpxA, not only showed the expected biophysical properties but also largely retained their inherent natural molecular functions, including autokinase activity, phosphotransferase activity, phosphatase activity, and the activity of signal receptors involving water‐soluble transmembrane domains. These results indicate that the QTY code can effectively enhance protein solubility while maintaining their biological activity and function.

### Enzyme activity engineering

Natural enzymes often fail to meet the demands of industrial or laboratory settings in terms of catalytic efficiency, necessitating molecular modifications to enhance their specific properties and better satisfy practical application requirements. By modifying enzyme molecules, not only can their catalytic efficiency for specific substrates be improved, but they can also be tailored to better suit different substrates or to specifically target one substrate without affecting others. Additionally, enzyme engineering can help reduce or eliminate undesired side reactions, ultimately increasing the purity and yield of the target product. This is particularly crucial for improving productivity and reducing production costs.

In recent years, various computational tools and techniques have been developed for enzyme activity engineering. The aforementioned DLKcat is the most famous tool for predicting changes in enzyme activity. Another tool, UniKP^140^, predicts enzyme kinetic parameters, including *K*_cat_, *K*_m_, and *K*_cat_/*K*_m_. This framework integrates multiple machine learning algorithms and feature selection methods, significantly improving the accuracy of enzyme kinetic parameter predictions and providing important reference data for enzyme engineering. Additionally, CPDiffusion^141^ is a diffusion probability model framework that can generate diverse new sequences for proteins with specific functions by integrating backbone structures, active sites, and other generating conditions. This method learns the implicit mapping rules between protein sequences, structures, and functions at a very low model training and data cost. The generated protein sequences, when validated through wet experiments, have shown significant improvements in DNA cleavage activity, even surpassing the activity of any known mesophilic wild‐type protein. Compared to traditional directed evolution methods, CPDiffusion can modify hundreds of amino acids in a single step, offering new possibilities for enzyme activity engineering.

The CPDiffusion team generated 27 new artificial KmAgos (Km‐APs) and 15 artificial PfAgos (Pf‐APs). Compared to the template wild‐type (WT) proteins, these engineered enzymes share 50% to 70% sequence identity. In comparison to other WT proteins in NCBI (excluding the template), the sequence identity of the APs is less than 40%. Unlike traditional rational design methods, the entire process of model training and inference requires almost no expert guidance and can automatically identify highly conserved regions. This allows for more modifications in non‐conserved regions while ensuring functionality, ultimately increasing the diversity of the generated sequences.

### Enzyme affinity engineering

Enzyme affinity engineering is a key approach for enhancing catalytic efficiency and application performance. Natural enzymes may show low substrate affinity under specific conditions, limiting their use in industries, medicine, and other fields. Optimizing enzyme affinity can enable enzymes to catalyze reactions efficiently even at low substrate concentrations, significantly improving their performance. Enzyme affinity is typically represented by the *K*_m_ value, with a lower *K*_m_ value indicating a higher binding ability to the substrate.

In recent research, the EnzyGen model^142^ has introduced a new attention and substrate crossover network for designing enzymes with good folding structures and high enzyme–substrate binding capabilities^98^. This model uses a joint training objective that includes sequence generation loss, position prediction loss, and enzyme–substrate interaction loss. Experimental results showed that when tested on 3157 enzyme families from the EnzyBench dataset, EnzyGen consistently outperformed all other models in the 323 test families, particularly in substrate binding affinity, surpassing the best baseline model by 10.79%. These findings not only demonstrate the superior performance of EnzyGen in enzyme affinity engineering but also provide new ideas and tools for designing high‐affinity enzymes in the future.

## CHALLENGES

AI technologies have demonstrated their important role in enzyme discovery, design, and engineering. However, to further advance the field of molecular retrosynthesis, these methods still face numerous challenges, which are discussed below.

### Challenges in molecular retrosynthesis route planning

Retrosynthesis is a critical component of biochemical pathway design, but it still encounters many challenges in practical applications.

#### Acquisition of molecular substructures

Current methods rely on SMILES pair encoding to obtain molecular substructures. However, the substructures generated by this method are often chemically uninterpretable and susceptible to the influence of fragment size. Therefore, more advanced fragmentation methods are needed to obtain more robust and chemically interpretable molecular substructures^143^. Combining SMILES pair encoding with modern sequence alignment techniques can provide more reasonable substructure information for retrosynthesis.

#### Determination of reaction site activity

There are deficiencies in determining the reactivity of different reaction sites, leading to the generation of unreasonable and infeasible reaction pathways. To address this issue, chemical modules can be introduced during the decoding process to guide the generation of effective and feasible reactions^144^. These chemical modules, based on established chemical reaction mechanisms and experimental data, can enhance the accuracy of retrosynthesis predictions.

#### Integration of reaction class knowledge

Reaction class information plays a crucial role in retrosynthesis prediction, but integrating it effectively into the model remains a significant challenge. Reaction class label embeddings can be input as hard constraints during the decoding process or different reaction class labels can be used as prompts to enhance the diversity of predictions^145^. Reaction class label embeddings ensure that the generated reaction pathways are chemically logical. In addition, different reaction class label prompts can expand the model's exploration range, resulting in a wider variety of reaction pathways.

#### Substrate and product atom matching

Accurate matching of substrate and product atoms is a key step in retrosynthesis prediction and is particularly important for constructing large training datasets. By improving atom matching algorithms, the accuracy and efficiency of retrosynthesis predictions can be significantly enhanced^146^. For instance, the Z‐align algorithm, which considers chemical and structural similarity of reference molecules, shows a high success rate for docking molecules (with a root mean square deviation of less than 2 Å) and can also be used for high‐precision atom mapping in reactions^146^.

#### Designing multistep retrosynthesis routes from single‐step retrosynthesis

Most current retrosynthesis methods focus on single‐step reactions, and their extension to complete retrosynthesis route design is a pressing issue. For example, Monte Carlo Tree Search (MCTS), which includes four stages consisting of selection, expansion, simulation, and backpropagation, is an effective method for synthetic planning^147^. Additionally, beam search strategies, such as hypergraph exploration strategies, can be used in retrosynthesis path search algorithms. This strategy considers multiple optimal options based on beam size to expand the path search tree^148^. Another method is A* search, such as the Retro* algorithm, which is a best‐first search algorithm capable of rapidly expanding the most promising precursors, thereby improving search efficiency and accuracy^149^.

### Enzyme diversity and unexploited potential of enzymes

Although humans have discovered and studied thousands of enzymes, a vast number of enzymes and metabolic pathways remain unexplored in nature. These uncharacterized enzymes may catalyze nonnatural chemical reactions, such as the introduction of fluorine groups, the formation of nitro compounds, and others, providing potential for the generation of novel functional groups^150^. For example, functional groups like nitro, cyano, and phosphonate can be achieved through biocatalysis^150^, ^151^. Additionally, the introduction of rare elements such as fluorine, arsenic, and selenium offers new methods for drug and functional material synthesis, further expanding the scope and capabilities of chemical synthesis^152^.

Several strategies are used to discover these enzymes with untapped potential.

#### Genome mining

Through genome data analysis, potential enzyme genes can be identified, and their functions can be predicted, which can be used for developing new reactions. This strategy typically relies on metabolic pathway databases (such as KEGG) and various bioinformatics tools, providing strong support for the discovery and functional characterization of new enzymes^13^. Advanced genome mining techniques not only identify homologous genes of known enzymes but also discover enzymes with novel functions^153^.

#### High‐throughput screening

Standardized and automated screening systems can rapidly test the reactivity of thousands of enzymes, significantly accelerating the discovery of novel enzymes^154^. Combining microfluidics and droplet screening technologies can substantially reduce screening costs and improve efficiency^155^. These technologies enable researchers to evaluate a large number of enzymes in a short period of time, identifying those with the desired catalytic activity, which provides powerful tools for the development of new reactions. This ultimately facilitates the automation of the “design‐build‐test‐learn (DBTL)” cycle in synthetic biology research, enhancing the efficiency of studies in both fundamental and applied areas. Examples of such platforms include the iBioFAB platform at the University of Illinois at Urbana‐Champaign, the EGF platform at the University of Edinburgh, the automated platforms at the Tianjin Institute of Industrial Biotechnology of the Chinese Academy of Sciences (TIB‐CAS), the iBioFoundry at Zhejiang University, and the Shenzhen Biofoundry at the Shenzhen Institutes of Advanced Technology of the Chinese Academy of Sciences (SIAT‐CAS)^155^.

#### LLMs

With the emergence of the new generation of ChatGPT, LLMs can more efficiently and intelligently mine existing enzyme data from patents, literature, and public databases.

### Challenges in de novo enzyme design

In recent years, significant progress has been made in methods for de novo enzyme design. However, these methods still face certain limitations and challenges. This progress and its associated challenges further underscore the importance of genome mining and high‐throughput screening, as well as the need for new technologies and approaches to address them. Current methods heavily rely on the EC classification. Although EC classification prediction has witnessed significant advancements in the deep learning era^11^, ^156^, and generative models can produce enzyme sequences similar to reference sequences and achieve the desired EC classification^126^, these advancements still have limitations. The primary reason is that designing enzymes solely based on EC classification restricts the generative models’ ability to generalize to new and unseen reactions^157^.

Additionally, a major challenge in de novo enzyme design using current models is their limited analysis of enzyme–substrate catalytic mechanisms. Even if new enzyme sequences can correctly fold into three‐dimensional structures, the catalytic pockets and the complex binding interactions between the enzyme and substrate are often overlooked or remain unclear. Fortunately, a recent framework called GENzyme has been proposed to tackle these challenges^157^. GENzyme not only generates enzymes that can catalyze novel reactions but also addresses the key issue of enzyme–substrate interactions by generating their binding structures. This advancement offers new insights into de novo enzyme design and is expected to play a significant role in future enzyme design and protein engineering.

### Challenges in expanding biocatalysis to the chemical space through enzyme engineering

Biocatalysis, a distinctive chemical technology, has significantly expanded the chemical space by incorporating enzyme analysis, showing great potential in drug discovery and synthesis. The realization of this potential relies not only on improved molecular retrosynthesis route planning and the discovery of new enzymes but also on further enhancement of enzyme functions through enzyme engineering. Several key aspects demonstrate how biocatalysis extends the chemical space.

#### Generation of highly sp^3^‐rich complex structures

Biocatalysis can generate molecules with complex three‐dimensional structures through enzyme‐catalyzed reactions such as cyclization, rearrangement, and carbon backbone reorganization^158^, ^159^, ^160^. For example, ene‐reductases and cytochrome P450 enzymes trigger cyclization through oxidation reactions, enabling the rapid construction of stereocomplex molecules^161^, ^162^. Additionally, biocatalysis can produce complex chiral molecules from simple substrates (such as alcohols, aldehydes, and ketones) in a single step, further enriching the chemical space.

#### Inspiration from natural products

The chemical diversity of natural products far exceeds that of synthetic compounds, often featuring more chiral centers and complex three‐dimensional structures^163^, ^164^. Biocatalysis, which mimics the biosynthetic pathways of natural products, can rapidly generate molecules that are similar to natural products^165^. For instance, expanding the diversity of terpenoids or polyketides through enzyme engineering can aid in the synthesis of new functional analogs of natural products^166^.

#### Diversity‐oriented synthesis (DOS)

DOS is a strategy that combines the complexity of natural products with the flexibility of synthetic chemistry^167^. By leveraging the core structures of natural products and using enzyme catalysis to generate diverse molecular fragments, this strategy provides new pathways for drug development^168^ DOS enables the rapid generation of a large number of structurally diverse compounds, improves synthetic efficiency and selectivity, and brings significant advancements to the discovery and optimization of new drugs^169^.

## CONCLUDING REMARKS

Despite the significant advantages gained by the high selectivity of enzymes in biocatalysis, this selectivity also leads to a high dependence on specific substrates, which limits their application range to some extent. Moreover, the cost of enzyme development and optimization is relatively high, especially in industrial‐scale applications, where issues of enzyme stability and efficiency still need to be further addressed. To overcome these challenges, future research should focus on gaining a deeper understanding of enzyme catalytic mechanisms and acquiring more knowledge about enzyme catalysis and potential reaction ranges through the integration of experimental and computational models.

The development of bioinformatics tools is also critical. Comprehensive bioinformatics tools can integrate genomics, metabolomics, and reaction databases to predict the substrate range, reaction efficiency, and possible pathways of enzymes. Data sources for model training will include two aspects: (i) “legacy data” from patents, literature, and public databases, and (ii) “new data” generated by standardized, automated, and high‐throughput technologies. Trained models can also continuously interact with high‐throughput facilities (DBTL) for iterative optimization through a reinforcement learning mode. The Registry and Database of Bioparts for Synthetic Biology (RDBSB) platform is a typical example^170^, used for collecting, storing, and sharing detailed qualitative and quantitative data of catalytic biological components. This platform aggregates over 80,000 catalytic biological components with experimental evidence from public resources through manual curation and literature mining. The RDBSB platform places particular emphasis on collecting and organizing experimental conditions such as optimal pH, temperature, and compatible chassis, and encourages the submission of new catalytic biological components to continually enrich the resource with experimentally validated data.

The extensive application of AI will also provide strong theoretical support for the directed evolution and functional prediction of enzymes^171^. Through the analysis of structural and functional data of enzymes utilizing deep learning, it is possible to accurately predict potential catalytic reactions and substrate specificity, offering new insights for enzyme design and optimization^157^.

In summary, despite facing numerous challenges, the development and optimization of enzymes will benefit from rapid advancements in biotechnology, computational tools, and materials science, expanding the application prospects of biocatalysis in drug development, green chemistry, and the synthesis of complex molecules.
